# Conformation Study of Dual Stimuli-Responsive Core-Shell Diblock Polymer Brushes

**DOI:** 10.3390/polym10101084

**Published:** 2018-09-30

**Authors:** Kaimin Chen, Lan Cao, Ying Zhang, Kai Li, Xue Qin, Xuhong Guo

**Affiliations:** 1 College of Chemistry and Chemical Engineering, Shanghai University of Engineering Science, Shanghai 201620, China; 18301939658@163.com; 2 State Key Laboratory of Chemical Engineering, School of Chemical Engineering, East China University of Science and Technology, Shanghai 200237, China; lancaoecust@outlook.com (L.C.); zy12fearless@163.com (Y.Z.); qinxue384@163.com (X.Q.)

**Keywords:** stimuli-response, polymer brush, polyelectrolyte, bovine serum albumin

## Abstract

Stimuli-responsive nanoparticles are among the most popular research topics. In this study, two types of core-shell (polystyrene with a photoiniferter (PSV) as the core and diblock as the shell) polymer brushes (PSV@PNIPA-*b*-PAA and PSV@PAA-*b*-PNIPA) were designed and prepared using surface-initiated photoiniferter-mediated polymerization (SI-PIMP). Moreover, their pH- and temperature-stimuli responses were explored by dynamic light scattering (DLS) and turbidimeter under various conditions. The results showed that the conformational change was determined on the basis of the competition among electrostatic repulsion, hydrophobic interaction, hydrogen bonding, and steric hindrance, which was also confirmed by protein adsorption experiments. These results are not only helpful for the design and synthesis of stimuli-responsive polymer brushes but also shed light on controlled protein immobilization under mild conditions.

## 1. Introduction

In recent years, core-shell nanoparticles have received increasing interest. As core-shell structure nanoparticles, spherical polymer brushes are formed by attaching one end of the polymer chain to a core surface. Spherical polymer brushes have good stability, wettability, stimuli responsiveness, and so on [1,2,3,4,5]. Therefore, they have been widely used in the field of sensors [6,7,8], drug loading [9], protein separation [10], antifouling materials [11,12], nano-metal catalysis [13], and Pickering emulsion [14]. At present, the most widely used stimuli-responsive polymer brushes have one or more stimuli responses, such as pH [6,15], salt concentration [16], temperature [5,17], light [18], and magnetic response [4,19]. At present, the research on single-response polyelectrolyte brush theory and applications is considerably mature. In particular, pH-responsive brushes and temperature-responsive brushes have received widespread attention because of their ease of regulation.

In the case of pH-responsive polyelectrolyte brushes, the aggregation and dispersion can be controlled by changing the pH of the system, and the conformation of the particles. As a carrier, nano-sized metal particles can be reduced and regulated with polyelectrolyte brushes to provide an excellent catalyst system for organic synthesis [20,21]. It is also possible to control the adsorption and desorption of proteins, such as bovine serum albumin (BSA), with a change in pH, which enables the controlled separation of proteins [22,23,24]. However, there are still problems for controlled protein separation. It is difficult to separate protein-loaded polyelectrolyte brushes from a protein solution, particularly when the particle size is small.

Temperature-responsive polymer brushes are widely studied as temperature sensors, as their size and shape change with a change in the system temperature. Moreover, the catalytic activity of a metal catalyst system supported with a temperature-sensitive polymer brush can be controlled by the regulation of the system temperature [25]. Zeid et al. regulated the lower critical solution temperature (LCST) of a polymer brush by controlling the brush-layer monomer ratio and achieved the controlled release of the anticancer drug DOX by adjusting the system temperature, which provides a prospect for the targeted release of drugs [26]. However, when the proteins were loaded, temperature-sensitive polymers mostly combined with the proteins because of their hydrophobic properties, posing the problem of reduced loading.

Double-responsive nanoparticles can compensate for the disadvantages of single-responsive nanoparticles to some extent. For example, when we consider both the pH and the temperature response, we can use the pH-responsive segment to increase the drug loading rate and the temperature response for a controlled release [27,28]. This combination offers further advantages in biomedical applications.

Thus far, considerable research has been conducted on polymer brushes that combine temperature and pH responses. PS@PDMAEMA with the *N*,*N*′-dimethylaminoethyl methacrylate (DMAEMA) monomer grafted onto the polystyrene (PS) core surface has both pH- and temperature-response characteristics [29]. The copolymerization of the DMAEMA monomer and the *N*-isopropyl acrylamide (NIPA) monomer in different ratios can yield different LCSTs [30]. Cang et al. explored the temperature-sensitive performance of the copolymerization brush polystyrene@poly(*N*-isopropyl acrylamide)-*co*-poly(acrylic acid) (PS@PNIPA-*co*-PAA) with different acrylic acid (AA) additions under different pH values [31]. However, these polymer brush chains are randomly copolymerized with structural uncertainties and uncontrollability present the problem of a “loss” of thermo-sensitivity, even when a small amount of AA is added [31,32]. Therefore, living/controlled radical polymerization has become the first choice for the synthesis of polymer brushes. Atom transfer radical polymerization (ATRP), reversible addition-fragmentation chain transfer (RAFT), nitroxide-mediated polymerization (NMP), and iniferter are commonly used for living radical polymerization [33]. Linear block polymers prepared by living polymerization can be self-assembled into the core-shell structure under the external pH or temperature stimulation to achieve the controlled release of drugs [34,35,36,37]. The core-shell structure of spherical polymer brushes are usually synthesized by ATRP. Shi et al. grafted block poly(*N*-isopropyl acrylamide)-*b*- poly(2-succinyloxyethyl methacrylate) (PNIPA-*b*-PSEM) on the gold surface through ATRP; the nanoparticles not only showed good temperature sensitivity and pH responsiveness but also showed photo-induced thermosensitivity because of the presence of the gold nucleus [18]. Wu et al. grafted two-block poly(*N*-isopropyl acrylamide)-*b*-poly(4-vinylpyridine) (PNIPA-*b*-P4VP) double-responsive brushes from silica cores by ATRP [38] and found that temperature-sensitive performance declined because of the impact of the P4VP segment. In addition, the difficulty of removing the transition metal catalyst in the process of ATRP limits its application in biomedicine. In contrast, the iniferter method has the advantages of mild experimental conditions, simple operation, and a wide range of applications. Thus, it has greater application advantages in biomedicine. The photoiniferter method is widely used because it is convenient to control the “start” and “stop” of polymerization by turning the UV lamp on and off, respectively. Sakiko et al. prepared a two-block polymer brush with PNIPA and PNIPA-*co*-PAA by using the photoiniferter method, studied the effect of a small amount of added AA on its thermo-sensitivity and explored their differences with respect to protein adsorption [39,40]. Imroz et al. then prepared a variety of diblock copolymer brushes by using the photoiniferter method [41]. Nataliya designed and synthesized a variety of multi-shell nanoparticles by combining the heat-induced and photoiniferter methods [42].

**Scheme 1:**
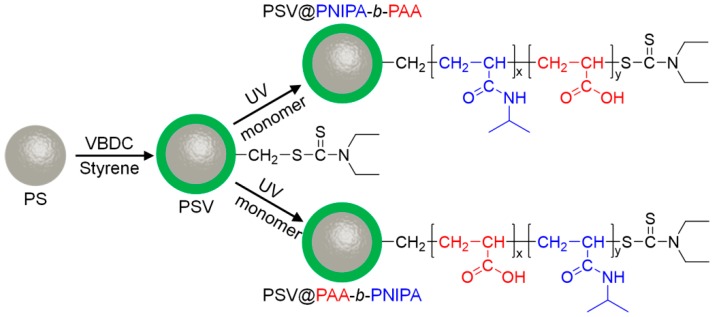
Schematic representation of the synthetic process and chemical structures of diblock polymer brushes.

In this study, we designed a spherical dual-responsive diblock polymer brushes PSV@PNIAP-*b*-PAA and PSV@PAA-*b*-PNIPA by using surface-initiated photoiniferter-mediated polymerization [12] on the surface of PS cores with the controlled polymerization of the NIPA and AA monomers (as shown in Scheme 1). The difference in the stimulus response, particularly the temperature response of the different structures, was explored. And conformational change was also studied on the basis of protein adsorption.

## 2. Experimental

### 2.1. Materials

Styrene (St, 99%) and acrylic acid (AA, 99%) were purchased from Lingfeng Chemical Reagent Co., Ltd. (Shanghai, China) and purified by distillation under a reduced pressure to remove the inhibitors. *N*-isopropyl acrylamide (NIPA, 98%) was purchased from Aladdin Industrial Corporation (Shanghai, China) and recrystallized from hexane. Sodium dodecyl sulfate (SDS, 99%) was purchased from Amresco, Inc. (Boise, ID, USA) K_2_S_2_O_8_ (KPS, 99%) and bovine serum albumin (BSA) (98%) were purchased from J&K Chemical (Shanghai, China). Photoiniferter 4-vinylbenzyl *N*,*N*-diethyldithiocarbamate (VBDC) was synthesized from 4-vinybenzyl chloride (J&K Chemical, 99%) and sodium diethyldithiocarbamate (J&K Chemical, 99%) as described in Otsu’s work [43]. The water used for all the experiments was purified using reverse osmosis (Millipore Milli-RO, Merck KGaA, Darmstadt, Germany) and subsequent ion exchange (Millipore Milli-Q, Merck KGaA, Darmstadt, Germany). All the other reagents of analytical grade were purchased from Lingfeng Chemical Reagent Co., Ltd. (Shanghai, China) and used as received.

### 2.2. Synthesis of Active PSV (PS Cores with Photoiniferter VBDC)

First, the PS cores were obtained from continental emulsion polymerization. A mixture of 0.04 g of SDS, 0.12 g of KPS, 2 g of St and 47.84 g of water was placed in a three-neck round-bottom flask equipped with a mechanical agitator. After stirring (300 rpm) the mixture for 1 h under nitrogen protection, the polymerization was started by increasing the temperature to 80 °C; it lasted for 1 h. Then, the obtained PS cores were purified by ultrafiltration. PS cores with photoiniferter VBDC (PSV) were prepared by seeded soap-free emulsion copolymerization [44]. In general, 15.5 g of the PS core emulsion (containing 0.5 g of solids), 0.05 g of KPS, and 200 g of water were taken in a three-neck round-bottom flask equipped with a mechanical agitator. The mixture was gently stirred for 0.5 h (100 rpm) under nitrogen, and then the temperature was increased to 70 °C. Once the temperature reached 70 °C, the monomer mixture (VBDC (0.2 g), St (0.8 g)) was continuously introduced into the reactor at the stirring speed of 300 rpm. After the monomer addition, the polymerization was further continued for 5 h. The resulting PSV was purified by ultrafiltration.

### 2.3. Synthesis of Diblock Polymer Brushes (PSV@PNIPA-b-PAA, and PSV@PAA-b-PNIPA)

The PSV@PNIPA-*b*-PAA nanoparticles were synthesized by surface-initiated photoiniferter-mediated polymerization (SI-PIMP). Initially, the PNIPA polymer layer was grafted from the surface of the PSV particles by UV irradiation. Then, 0.5 g of the NIPA monomer, 75 g of the PSV emulsion (containing 0.5 g of solids) and 14.5 g of water were added to the photo-reactor while gently stirring the mixture, and then, the reaction mixture was UV-irradiated for 2 h under nitrogen at ambient temperature. The resulting nanoparticles of PSV@PNIPA were purified by ultrafiltration. Hereafter, the PAA polymer layer was grafted from end of the PNIPA chains to obtain PSV@PNIPA-*b*-PAA. Typically, 0.5 g of the AA monomer, 35 g of the PSV@PNIPA emulsion (containing 0.5 g of solids), and 14.5 g of water were UV-irradiated for 4 h. After the polymerization, the resulting particles of PSV@PNIPA-*b*-PAA were purified by ultrafiltration. The PSV@PAA-*b*-PNIPA nanoparticles were obtained by using a similar method with the reverse order of the NIPA and AA monomer addition.

### 2.4. Characterization

#### 2.4.1. Dynamic Light Scattering (DLS)

The hydrodynamic size and zeta potential of particles were performed by PSS Nicomp 380 (Particle Sizing Systems, Port Richey, FL, USA) with the measurement angle of 173° (backscatter), and collected by the autocorrelator. Samples were diluted to appropriate concentration and measured three times in different pH and temperature. Before the temperature test, the samples need to be equilibrated for 10 min.

#### 2.4.2. Turbidimetric Titration (Turbidimetry)

Transmittance (%*T*) was recorded with a Brinkmann PC 950 colorimeter (Brinkmann, Melbourne, FL, USA) (with a 420 nm filter), connected to a 20 mm path length probe. The turbidity was reported as 100-*T*%. All solutions were prepared with deionized water or phosphate buffer saline. In general, 0.1 mg of BSA and 0.02 mg of nanoparticles were putted into 25 mL of phosphate buffer (1 mM). The turbidity change with pH was monitored upon addition of 0.1 M of HCl to the system. Polymer brush-free blanks and protein-free blanks were subtracted in the data processing.

#### 2.4.3. Transmission Electron Microscopy (TEM)

The TEM images were performed by a transmission electron microscope JEOL-1400F (JEOL USA, Peabody, MA, USA) at 200 kV operating voltage. Samples were diluted to proper concentration by 1 mM PB, 10 μL of the sample solution was putted by pipettes to carbon grid and allowed to dry at room temperature.

## 3. Results and Discussion

### 3.1. Size of Core-shell Diblock Polymer Brushes

**Figure 1:**
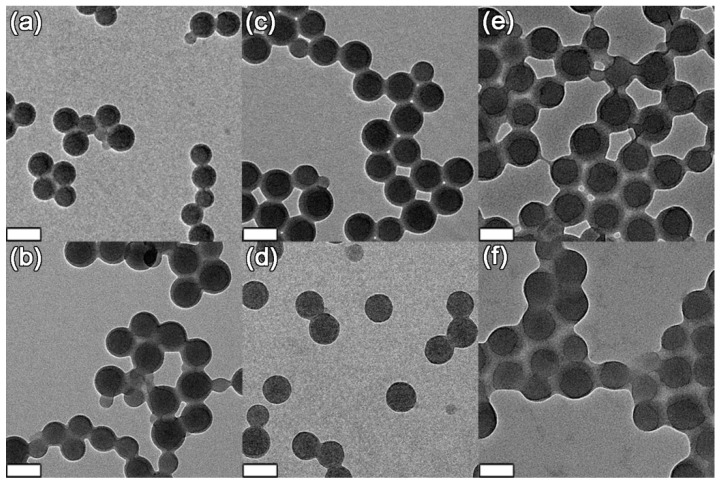
Transmission electron Microscopy (TEM) images of (**a**) PS, (**b**) PSV, (**c**) PSV@PNIPA, (**d**) PSV@PAA, (**e**) PSV@PNIPA-*b*-PAA, and (**f**) PSV@PAA-*b*-PNIPA. Scale bar = 100 nm.

Two types of diblock polymer brushes, i.e., PSV@PNIPA-*b*-PAA and PSV@PAA-*b*-PNIPA, were prepared using a previously reported method [45]. The sizes of various nanoparticles during the preparation were monitored by using both TEM and DLS characterization. First, TEM was used to observe the size change which is shown in Figure 1. The size of PS was found to be ~70 nm (Figure 1a), and the PSV particles were larger (Figure 1b), indicating that photoiniferter VBDC was successfully coated on the PS core. Fourier-transform infrared spectroscopy (FTIR) spectra (Figure S1) also confirm the successful coating and elemental analysis shows that the VBDC amount on PSV is around 7.5 mol% (Table S1). However, the size of PSV@NIPA (Figure 1c) or PSV@PAA (Figure 1d) was almost the same as that of PSV, which may be attributed to the relatively low grafting density of the first block and the drying polymer chains collapsing on the PSV, which was also observed in a previous report [11]. However, after the grafting of the second block, the size increased with a blurry shell outside and the nanoparticles tended to agglomerate into clusters. Elemental analysis confirmed the successful grafting of blocks and the monomer conversions of AA and PINIA are approximately 50% (Table S2). Polymer brushes were usually used in aqueous solutions such as protein or catalyst immobilizations. Their size change in the solution was very important for a detailed investigation and further application. DLS was then used to determine the size change for all the nanoparticles in an aqueous solution.

The DLS was carried out in 1 mM phosphate-buffered saline (PBS) with a pH value of 7. All the samples were tested in triplicates, and the mean value is shown in Table 1 with a standard deviation of less than 2 nm (DLS traces are shown in Figure S2). Similar to the TEM results, the size of PS (70 nm) increased to 100 nm of PSV. However, the size of the polymer brushes was obviously different between TEM and DLS. The hydrodynamic size of PSV@PNIPA and PSV@PAA increased to 160 and 180 nm with the first block thickness of 30 and 40 nm, respectively. Similarly, the apparent size of PSV@PNIPA-*b*-PAA and PSV@PAA-*b*-PNIPA increased to 330 and 210 nm with the second block thickness of 85 and 15 nm, respectively. The size of the second block of PAA was reasonable as PAA could stretch out because of the electrostatic repulsion at a pH value of 7. The second block of PNIPA was extremely small as compared with the NIPA dosage, which will be discussed further in the following sections.

### 3.2. Stimuli-Responsive Properties

#### 3.2.1. pH-Stimulus Response

**Figure 2:**
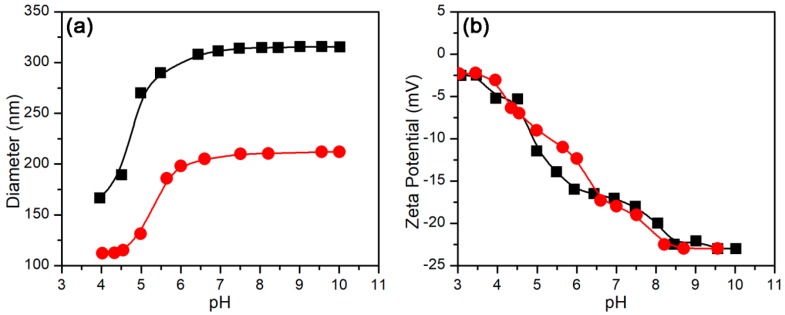
Effect of pH on (**a**) hydrodynamic diameter and (**b**) zeta potential of diblock polymer brushes. (■) PSV@PNIPA-*b*-PAA, (●) PSV@PAA-*b*-PNIPA.

The DLS and zeta potential information for different pH values of PSV@PNIPA-*b*-PAA and PSV@PAA-*b*-PNIPA showed that both exhibited a good pH-stimulus response (Figure 2). With an increase in the pH, their hydrodynamic diameters increased because of the deprotonation of the PAA chains. Most of the diameter changes were observed in the pH range of 5–7. The particle size range of PSV@PNIPA-*b*-PAA was 165–320 nm, while that of PSV@PAA-*b*-PNIPA was 110–210 nm. During the DLS experiment, we found that aggregation occurred when the pH value was less than 4 for both types of polymer brushes. Moreover, the smallest sizes before aggregation at the low pH values of the two polymer brushes were different, which was interesting. We observed that when the PAA block was located on the outer layer, the minimum particle size of the pH response was consistent with the particle size of PSV@PNIPA (160 nm), whereas when the PAA block was located in the inner layer, the minimum particle size of the pH response was close to that of PSV (100 nm). We also observed for both types of polymer brushes that when the pH was below 4, aggregates were found in the system, and the size sharply increased to several micrometers and could not be detected by the instrument. The stability of the nanoparticles in solution is generally related to its surface charge, and here, the zeta potential was monitored along with the size by using the same samples.

The zeta potential results are shown in Figure 2b. We observed that both zeta potential curves almost overlapped at all the pH values. Because PNIPA was neutrally charged, the zeta potential mainly exhibited the PAA block behavior. The zeta potential was negative in the entire pH range and decreased with a decrease in the pH value. When the pH value was less than 4, the absolute value of the zeta potential was lower than 5 mV, indicating that the surface charge was not sufficient for good stability, which corresponded to the aggregation phenomenon discussed above.

We concluded that both types of polymer brushes exhibited good pH stimulus responses. For PSV@PNIPA-PAA, the PNIPA block seemed to support the inner brush layer, and the PAA contraction did not affect the PNIPA conformation. However, for PSV@PAA-*b*-PNIPA, when the PAA collapsed at a lower pH, the PNIPA block “disappeared.” At least, the PNIPA block did not fully extend outside, which could explain the small size increase when the first block PNIPA was grafted to PSV@PAA. The real conformation of PNIPA in PSV@PAA-*b*-PNIPA would be further investigated under different conditions or methods.

#### 3.2.2. Temperature-Stimulus Response

Because of the presence of the PNIPA chains in the diblock nanoparticles, PSV@PNIPA-*b*-PAA and PSV@PAA-*b*-PNPAM were expected to respond to the temperature stimulus. However, the PAA block and structure might affect their temperature responses. Thus, the size of the diblock polymer brushes was explored at various temperatures at a given ionic strength and pH value. PSV@PNIPA was first tested to observe its performance as a temperature stimulus (data not shown here), and the LCST was near 32 °C which was its intrinsic value.

**Figure 3:**
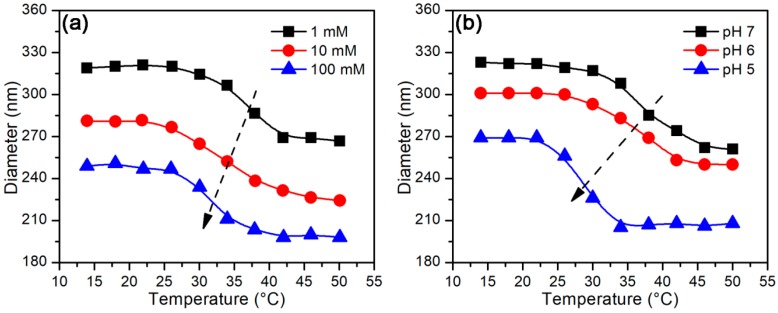
Effect of temperature on hydrodynamic diameters of PSV@PNIPA-*b*-PAA (**a**) in different ionic strength at pH of 7.4 and (**b**) at different pHs in 1 mM PBS.

Compared with PSV@PNIPA, the phase transition temperature range of PSV@PNIPA-*b*-PAA was relatively large and broad (22–46 °C), as shown in Figure 3, indicating that the outer layer PAA block prevented the coil-to-globule transition of the PNIPA block to some extent. Figure 3a shows that the phase transition diameter range in 1, 10 and 100 mM PBS was 320–270, 280–225 and 250–195 nm respectively; i.e., the size decreased with a decrease in the ionic strength over the entire temperature range [32]. Moreover, with an increase in the ionic strength, the LCST decreased a little, implying that the LCST leaned towards a high temperature at a lower ionic strength and recovered to its intrinsic value at a high ionic strength. At the pH of 7.4 and a low ionic strength of 1 mM, the PAA block was fully extended outside, as shown in Figure 2a, which might endow the extra hydrophilicity of diblock PNIPA-*b*-PAA and the contraction of PNIPA at a high temperature could be delayed by the hydrophilic PAA block. When the ionic strength increased, the PAA block became increasingly hydrophobic, and the intrinsic property of the PNIPA block then controlled the temperature response.

Another parameter affecting the PAA dimension was the pH value, and here, we selected the pH value at which the PAA dimension changed considerably. Figure 3b shows that the phase transition diameter ranges at pH 7, 6, and 5 were 320–265, 300–250 and 270–210 nm, respectively. Similarly, the size decreased obviously with a decrease in the pH, and the LCST became smaller at a low pH. However, a careful comparison of LSCT at a high pH with high ionic strength (triangle in Figure 3a) and that at a low pH with low ionic strength (triangle in Figure 3b) revealed some differences. We observed that the LCST was considerably low and the transition temperature range was very narrow in the case of a low pH and low ionic strength (Figure 3b). Furthermore, the size of PAA was very small at a low pH and high ionic strength, but the mechanism for the size change was different. At a low pH, the carboxyl group was protonated, and the repulsion between the PAA chains decreased, leading to a small size, while at high ionic strength, the carboxyl group remained deprotonated, but its charge was screened by the counterions. In other words, protonation had a considerable effect on the PNIPA contraction as compared to electrostatic screening.

**Figure 4:**
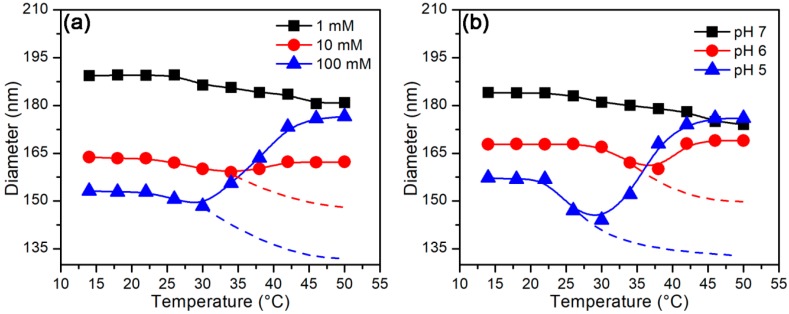
Effect of temperature on hydrodynamic diameters of PSV@PAA-*b*-PNIPA (**a**) in different ionic strength at pH of 7.4 and (**b**) at different pHs in 1 mM phosphate-buffered saline (PBS). Dashed lines represent the expected trends.

The temperature response of PSV@PAA-*b*-PNIPA was explored under the same conditions. Note that temperature response performance was considerably different from that of PSV@PNIPA-*b*-PAA, as shown in Figure 4. Both at low ionic strength (1 mM in Figure 4a) and a high pH (pH = 7 in Figure 4b), it exhibited a weak temperature response (~10 nm). In general, a shorter PNIPA chain exhibits a less apparent temperature response. It was very astonishing to find that at moderate ionic strength (10 mM) or pH (pH = 6), the size of the polymer brush fluctuated; i.e., first, it decreased a little and then went back to its original size. When relatively high ionic strength (100 mM) or low pH (pH = 5) was used, at a low temperature, we could still observe the LCST phenomenon; i.e., the size decreased with the temperature, but when the temperature exceeded 30 °C, we observed an apparent upper critical solution temperature (UCST) phenomenon; i.e., the size increased with an increase in temperature. This abnormal behavior should be mostly ascribed to the PNIPA block because PAA is inert to temperature.

It was reported that there might be a certain interaction between the carboxyl group in PAA and the amide group in PNIPA because of the hydrogen bonding [46]. We then simply mixed PSV@PAA and PSV@PNIPA and found that the size could hardly be detected from DLS and the mixture turned turbid; even the sediment was observed after the mixture was left standing for a long time. Therefore, unlike the random copolymer of PAA and PNIPA, the diblocks of PAA and PNIPA could interact with each other under suitable conditions via hydrogen bonding. In the case of PSV@PAA-*b*-PNIPA, when the second PNIPA block was grafted from the PAA block, the newly generated PNIPA chains did not extend outward because the steric hindrance was not sufficiently strong for the out layer PNIPA stretching. Instead, the PNIPA block inversely moved into the PAA block layer and “bound” to the PAA chains via hydrogen bonding. Then, we could explain the abnormal phenomena observed for the diblock polymer brushes. The generation of the PNIPA block did not result in a considerable size increase of the second block (L2 in Table 1) because most of the PNIPA was entrapped in the PAA inner layer. Moreover, the combination of the PNIPA block with the PAA block decreased the electrostatic repulsion between the PAA chains. Both the pH stimulus response of PAA and the temperature stimulus response of PNIPA weakened after the combination of the PAA and the PNIPA blocks. This was the reason for the weak temperature response of PSV@PAA-*b*-PNIPA shown in Figure 4.

The “strange” temperature response of PSV@PAA-*b*-PNIPA could also be explained. At low temperatures (<30 °C), with the increase in the ionic strength and decrease in pH, the PAA block shrunk with the combined PNIPA block because of the presence of hydrogen bonding. Similarly, when the temperature increased, the PNIPA block contracted together with the PAA block. However, as the temperature continued to rise (>30 °C), the hydrogen bonding gradually disappeared. On the one hand, the PNIPA block was pulled out from the PAA block to increase the particle size. On the other hand, the previously constrained PAA stretched outward after gaining the electrostatic repulsion to increase the size. Both the pulling out of PNIPA and the stretching of PAA were advantageous for size increase.

**Scheme 2:**
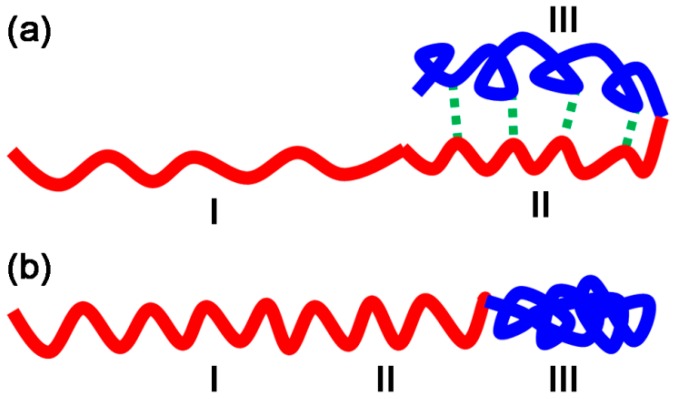
Conformation of PSV@PAA-*b*-PNIPA at high temperature under (**a**) 1 mM ionic strength or pH of 7 (**b**) 100 mM ionic strength or pH of 5.

To observe the competition between electrostatic repulsion, steric hindrance, and hydrogen binding more clearly, we drew a cartoon (Scheme 2) to explain the almost same size of PSV@PAA-*b*-PNIPA at the ionic strength of 1 and 100 mM (Figure 4a) or at pH 7 and 5 (Figure 4b) at 50 °C. At the low ionic strength or the high pH, there was strong hydrogen bonding between the PAA end and PNIPA. Most of the PAA block (part I) freely stretched out, but the end of the PAA block (part II) interacted with PNIPA via hydrogen bonding. On the one hand, this part of PAA could not fully stretch; on the other hand, the collapse of PNIPA at a high temperature was retarded by PAA. Hence, the temperature response was very small under these conditions. In contrast, when the high ionic strength or the low pH was used, the PAA block would collapse, and the hydrogen bonding was broken. The result was that PNIPA “escaped” from the PAA block and collapsed more densely without the support of PAA, and Part I and Part II of PAA showed the same contracted conformation status. Based on abovementioned complicated but reasonable mechanism, the apparent size of the polymer brushes could be the same even under different conditions.

### 3.3. Protein Adsorption Behaviors

#### 3.3.1. Effect of pH

To further verify the structure, we investigated the performance of protein adsorption under different conditions because polymer brushes could immobilize the protein to some extent. BSA was then introduced to interact with the polymer brushes. It has been reported that BSA adsorption in spherical PNIPA brushes is considerably small [39]. Moreover, the existing literature even shows that flat PNIPA brushes have anti-adsorption characteristics for human serum albumin [28]. In the case of low protein adsorption, there is no suitable method to monitor the adsorption amount in particle systems. Therefore, we used a high-resolution turbidimeter, which could respond to tiny conformational changes, accompanied by DLS to monitor the turbidity and size change under various conditions for a mixed system.

**Figure 5:**
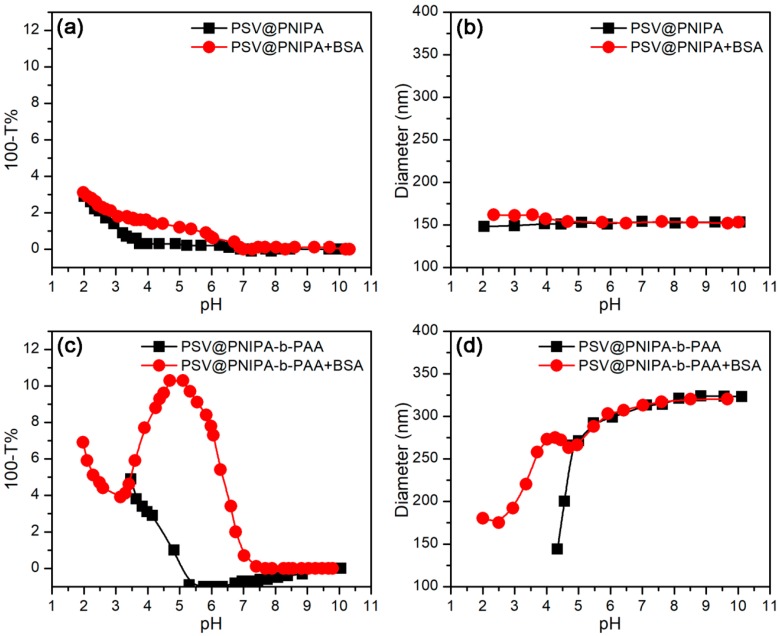
Effect of pH on protein adsorption in PSV@PNIPA and PSV@PNIPA-*b*-PAA via (**a**,**c**) turbidity and (**b**,**d**) hydrodynamic diameter change.

First, we studied the BSA adsorption of PSV@PNIPA (Figure 5a,b). The turbidity and diameter data exhibited little change with respect to those of PSV@PNIPA-*b*-PAA (Figure 5c,d). It was found that at pH values of higher than 6, no changes in the turbidity and size were observed, implying that there was no adsorption between BSA and the polymer brush. However, when the pH was less than 6, both the turbidity and the size increased a little, indicating that BSA immobilized in the polymer brush. However, the size increase was ~10 nm, which was similar to the size of the protein itself, from which we could assume that BSA was adsorbed on the PS core surface by the negatively charged surfactant. Therefore, the BSA adsorption of diblock polymer brushes was attributed to the PAA block because of the electrostatic forces; in particular, the proportions of the PAA block in the diblock nanoparticles were relatively high.

When the PAA block was grafted on PSV@PNIPA, the BSA adsorption increased obviously (Figure 5c,d). Because PNIPA almost adsorbed the “zero” protein, the size change after the BSA adsorption of PSV@PNIPA-*b*-PAA was ascribed to the PAA block, as shown in Figure 5d. No size change was observed at pH values higher than 5, while when pH was below 5, the size first increased a little (280 nm) and then decreased to a plateau (180 nm). At pH values lower than 5, all the sizes were larger than the size of the polymer brush without the protein. This was the typical protein adsorption behavior for spherical PAA brushes [24]. The size increase could be simply explained by the electrostatic interaction as BSA was positively charged below its isoelectric point (IEP) of 5.1. Turbidity data showed that the polymer brush started to interact with BSA at the pH of 7. At the pH of 5, the turbidity value reached a maximum and then began to drop until the pH of 3, and the turbidity value reached the minimum. The turbidity data provided more information about the protein adsorption behavior, particularly in the high pH range. In fact, BSA could adsorb in the polymer brush at a pH higher than its IEP; this is called the “wrong side” effect [47]. Here, the “wrong side” pH range was 5–7, in this range, the positive charge patches could interact with the negatively charged PAA segment.

**Figure 6:**
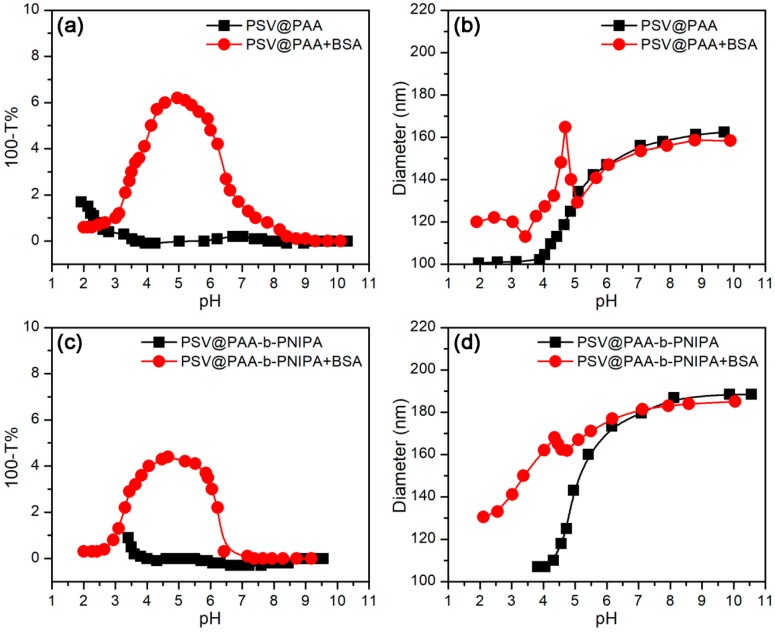
Effect of pH on protein adsorption in PSV@PAA and PSV@PAA-*b*-PNIPA via (**a**,**c**) turbidity and (**b**,**d**) hydrodynamic diameter change.

In contrast, we performed similar protein adsorption experiments for PSV@PAA-*b*-PNIPA and BSA. The results are shown in Figure 6. For PSV@PAA, its protein adsorption behavior was similar to that of PSV@PNIPA-*b*-PAA; i.e., there was a strong interaction at pH 2–8, as observed by the turbidimetry or at pH 2–5, as observed by DLS. When the second PNIPA block was grafted on PSV@PAA, we found that the overall size change reduced to some extent. The size of the fully extended polymer brush increased from 160 nm (PSV@PAA) to 190 nm (PSV@PAA-*b*-PNIPA) at pH 10 and the given ionic strength. We speculated in the abovementioned context that the grafted PNIPA block would interact with PAA block; therefore, most part of the PNIPA block would stretch inward resulting in a small size increase after grafting. When BSA adsorbed in the polymer brush near its IEP, the size variance for PSV@PAA was 35 nm (from 130 to 165 nm), but the value was only 10 nm (from 160 to 170 nm) for PSV@PAA-*b*-PNIPA. This mild change was also reflected in the turbidity, as shown in Figure 5c: That the maximum turbidity decreased from 6 to 4.5, indicating that the adsorbed protein reduced after PNIPA grafting. The reduced protein adsorption was attributed to the PAA segments which entangled with PNIPA. In other words, PNIPA grafting not only did not contribute but also reduced the protein adsorption to some extent.

#### 3.3.2. Effect of Temperature

Temperature affected the structural state of particles, which might, in turn, affect the protein adsorption. Thus, the BSA adsorption on the diblock polymer brushes at different temperatures would help to confirm the brush conformation. Because of the similar effects of the pH and the ionic strength on PAA stretching, three typical pH values were selected here instead of the ionic strength, and turbidimetry and DLS were used to monitor the interaction process with the temperature.

**Figure 7:**
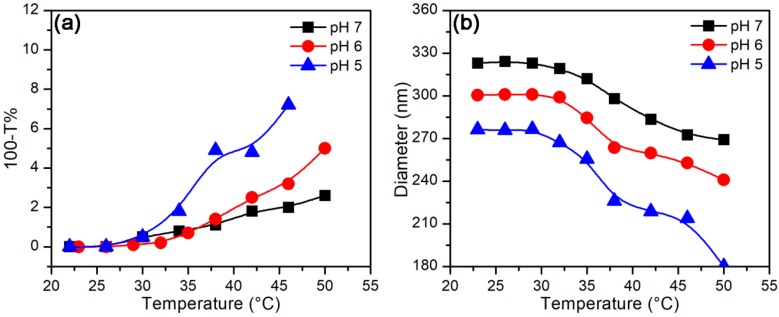
Effect of temperature on protein adsorption in PSV@PNIPA-*b*-PAA at various pH values via (**a**) turbidity and (**b**) hydrodynamic diameter change.

As shown in Figure 6, there were interactions between polymer brushes, but the size change was very small (less than 10 nm) at pH 5–7 for PSV@PNIPA-*b*-PAA. We found that the size change shown in Figure 7b was almost the same as that shown in Figure 3b at a low temperature (<40 °C), but when the temperature was relatively high, we observed that the size gradually declined without a plateau, as shown in Figure 3b. Furthermore, the declining trend was more obvious at a lower pH. As the temperature increased, the hydrogen bonds between PNIPA and water were destroyed, and the hydrophobicity of the PNIPA block increased, resulting in an increase in the protein adsorption of the PNIPA block, which caused the PNIPA block to become more compact. Moreover, the complex of the protein and the PAA block turned more hydrophobic and could interact with the inner PNIPA layer via hydrophobic forces, leading to a considerably smaller size at a high temperature. Figure 7a also demonstrates that the protein adsorption increased with an increase in the temperature, indicating that the hydrophobic PNIPA would adsorb more protein at a high temperature. Interestingly, we found a difference between DLS and turbidity. The turbidity increased, but the hydrodynamic diameter decreased with an increase in the pH at the same temperature. At a lower pH, more protein was adsorbed by PAA, and PNIPA began to adsorb some protein. The increased protein adsorption affected the light scattering and led to a higher turbidity of the mixed solution. However, DLS only reflected the apparent size change instead of the protein adsorption.

**Figure 8:**
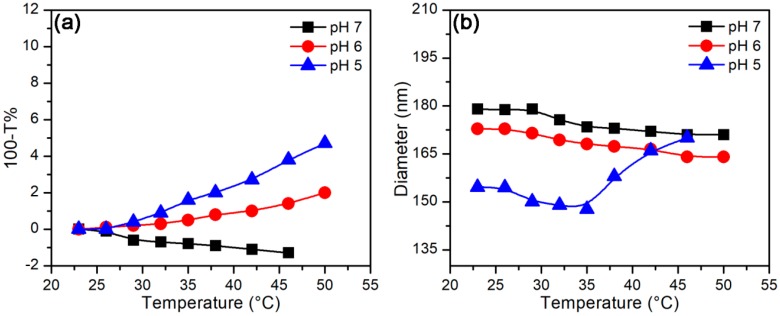
Effect of temperature on protein adsorption in PSV@PAA-*b*-PNIPA at various pH values via (**a**) turbidity and (**b**) hydrodynamic diameter change.

We then investigated the temperature effect on the protein adsorption in PSV@PAA-*b*-PNIPA. The results are shown in Figure 8. The size change trend was very similar to that of the pure diblock polymer brush shown in Figure 4. At pH 7, the size decreased a little, which was almost the same for the pure diblock polymer brush, but the case changed at pH 6 in that the size also gradually decreased a little, which was different from the trend shown in Figure 4b. It is known that the PNIPA block stretches inward at a low temperature at pH 6. When the temperature increased, PNIPA was released from PAA because of the lack of hydrogen bonding. Furthermore, the released PAA segments could turn to adsorb the protein and further compress the PAA block, leading to a size decrease instead of a size increase. The effect of protein adsorption on the size change can also be observed at the pH of 5: A mild size change occurred when the temperature increased as compared to the pure diblock polymer brush. This was the competition result between the PNIPA stretching out and the PAA segment compression after protein adsorption. From the turbidity results shown in Figure 8a, we also observed that the protein gradually increased with an increase in the temperature and at a higher turbidity and low pH. At the pH of 7, the turbidity decreased a little with temperature, because the turbidity change was dominated by the size decrease caused by the PNIPA contraction and the little protein adsorption did not contribute the turbidity.

## 4. Conclusions

In this work, pH- and temperature-sensitive diblock polymer brushes (PSV@PNIPA-*b*-PAA and PSV@PAA-*b*-PNIPA) were synthesized by using surface-initiated photoiniferter-mediated polymerization (SI-PIMP). We found that the conformational change of the polymer brushes was a result of the competition between electrostatic repulsion, hydrophobic interaction, hydrogen bonding, and steric hindrance. For PSV@PNIPA-*b*-PAA, the electrostatic repulsion dominated, and the PAA block was fully stretched in the outer layer. The normal ionic strength, pH, and temperature-stimuli response could be found, and LCST would shift to a lower temperature at a high ionic strength or a low pH value. For PSV@PAA-*b*-PNIPA, hydrogen bonding dominated and PNIPA entangled with the PAA segments, leading to a smaller size compared to PSV@PNIPA-*b*-PAA, and the hydrogen bonding-driven interaction was destroyed at an elevated temperature; then, PNIPA was released and stretched out because of steric hindrance. The competition among various interactions was also demonstrated by mixing these diblock polymer brushes with the BSA protein. The protein was mostly immobilized in the PAA block, but the relatively small protein adsorption by PNIPA at a low pH or a high temperature affected the conformational change of the diblock polymer brushes. This work is helpful for the design and the controlled synthesis of the stimuli-responsive diblock or triblock polymer brushes. The various conformations could be realized by changing the block component, length, or sequence. Furthermore, the protein immobilization could be tuned into a wider range under mild conditions via these types of polymer brushes, which would be useful in the biomedical field.

## Figures and Tables

**Table 1:** Size of diblock polymer brush during synthesis procedure.

Entry	PS/nm	PSV/nm	First block/nm	L1 ^1^/nm	Diblock/nm	L2 ^2^/nm
PSV@PNIPA-*b*-PAA	70	100	160	30	330	85
PSV@PAA-*b*-PNIPA	70	100	180	40	210	15

^1^ L1 denotes the thickness of the first block shell. ^2^ L2 denotes the thickness of the second block shell.

## Supplementary Materials

The following are available online at http://www.mdpi.com/2073-4360/10/10/1084/s1, Figure S1: FTIR spectra of (**a**) PS and (**b**) PSV, Figure S2: DLS traces of diblock polymer brushes. (**a**) PS; (**b**) PSV; (**c**) PSV@PNIPA; (**d**) PSV@PAA; (**e**) PSV@PAA-*b*-PNIPA; (**f**) PSV@PNIPA-*b*-PAA, Table S1: Elemental analysis of PS and PSV, Table S2. Elemental analysis of block polymer brushes and monomer conversions.
