# Supplementary material for: Conformation Study of Dual Stimuli-Responsive Core-Shell Diblock Polymer Brushes
Source: Polymers (Basel). 2018 Sep 30;10(10):1084. doi: 10.3390/polym10101084 (PMC6403727; doi:10.3390/polym10101084)
Supplement: Supplementary file 1 [file polymers-10-01084-s001.pdf]

Conformation study of dual stimuli-responsive core-shell diblock polymer brushes

Kaimin Chen [1,*], Lan Cao [2], Ying Zhang [2], Kai Li [1], Xue Qin [2] and Xuhong Guo [2,*]

[1] College of Chemistry and Chemical Engineering, Shanghai University of Engineering Science, Shanghai 201620, China; 1126953083@qq.com (K.L.)

[2] State Key Laboratory of Chemical Engineering, School of Chemical Engineering, East China University of Science and Technology, Shanghai 200237, China; 1572037177@qq.com (L.C.); zy12fearless@163.com (Y.Z.); 384229672@qq.com (X.Q.)

* Correspondence: kmchen@sues.edu.cn (K.C.); guoxuhong@ecust.edu.cn (X.G.); Tel.: +86-137-0172-0354 (K.C.); +86-137-6436-8083 (X.G.)

Table S1. Elemental analysis of PS and PSV.

	C%	H%	N%	S%	S/N[a]	S/N[b]
PS	92.31	7.69	-	-	-	-
PSV	87.20	-	0.95	4.19	4.40	4.57

[a] value from elemental analysis data

[b] theoretical value

Elemental analysis was carried on by an Elementar vario MICRO cube.

The molar ratio of VBDC and PS based on N% (0.95) and S% (4.19) is calculated as 7.07mol% and 8.24mol%, respectively. Averagely, the photoiniferter VBDC amount in PSV is ca. 7.5 mol%.

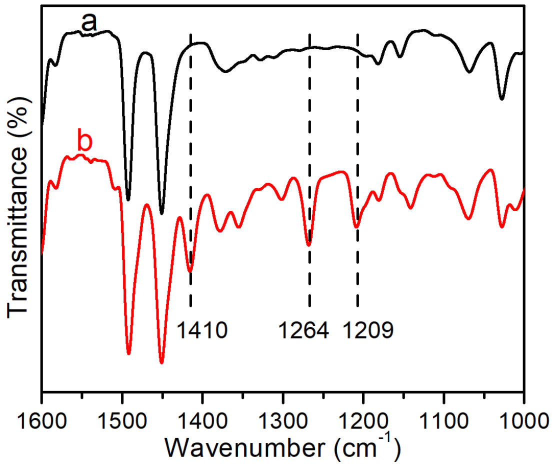

Figure S1. FTIR spectra of (a) PS and (b) PSV.

Peaks at 1410 cm-1, 1264 cm-1, and 1209 cm-1 are attributed to the stretching vibration of C=S, C-S, and C-N in photoiniferter VBDC, respectively.

Table S2. Elemental analysis of block polymer brushes and monomer conversions.

	C%	N%	S%	Conversion (%)
PSV	87.20	0.95	4.19	-
PSV@PNIPA	81.32	3.25	3.44	21.5
PSV@PNIPA-b-PAA	76.17	1.70	2.26	52.1
PSV@PAA	82.09	0.62	2.65	57.7
PSV@PAA-b-PNIPA	70.63	3.93	1.62	63.6

From Table S1, it is known that VBDC amount in PSV is 17.3wt% based on the S% data. Consequently, monomer conversions of AA and NIPA are calculated based on the S% data for different nanoparticles.

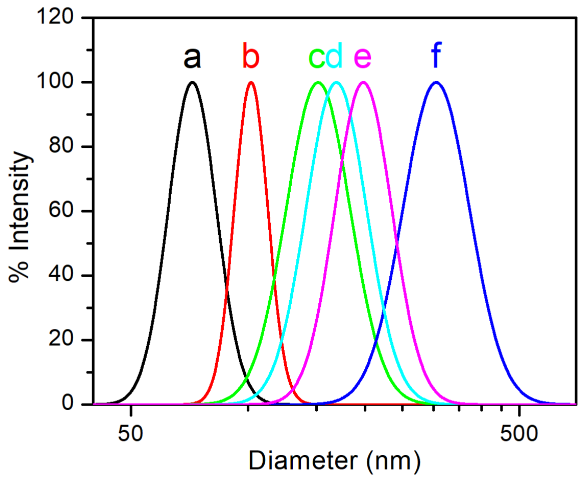

Figure S2. DLS traces of diblock polymer brushes. (a) PS; (b) PSV; (c) PSV@PNIPA; (d) PSV@PAA; (e) PSV@PAA-b-PNIPA; (f) PSV@PNIPA-b-PAA.
